# How Do Career Aspirations Benefit Organizations? The Mediating Roles of the Proactive and Relational Aspects of Contemporary Work

**DOI:** 10.3389/fpsyg.2018.02150

**Published:** 2018-11-09

**Authors:** Sabrine El Baroudi, Svetlana N. Khapova, Chen Fleisher, Paul G. W. Jansen

**Affiliations:** ^1^Department of Management and Organization, School of Business and Economics, VU University Amsterdam, Amsterdam, Netherlands; ^2^Utrecht School of Economics, Utrecht University, Utrecht, Netherlands

**Keywords:** career aspirations, organizational core competencies, taking charge, proactive behavior, networking

## Abstract

This paper examines how employees’ career aspirations benefit organizations, i.e., contribute to strengthening organizational capabilities and connections, by means of two aspects of contemporary work: proactive and relational. Data were collected from alumni of a public university in Amsterdam, the Netherlands, in two waves with a 1-year time lag. The results showed that employees with career aspirations strengthen: (a) organizational capabilities; and (b) organizational connections through their instrumental and psychosocial relationships. Interestingly, although employees’ career aspirations were positively associated with taking charge, we did not find that taking charge mediates the relationship between career aspirations and employees’ individual contributions to organizational capabilities. This study is the first to examine how individual career aspirations benefit organizations, and it discusses the results in light of their novel contributions to theory and practice.

## Introduction

Over the past decade, scholars have highlighted the importance of employees’ contribution to organizational effectiveness (e.g., Griffin et al., 2007; Strauss et al., 2015). This is because, in today’s dynamic and accelerated business environment, organizations rely heavily on employees’ novel and creative input to outperform competitors, excite customers and offer better services (Yang et al., 2016). Parallel to this trend, many career theorists contend that individuals have become active and agentic in their career development, generating strategies to fulfill their career needs and attain their career goals (Briscoe et al., 2006; Sullivan and Baruch, 2009). Thus, while managers are exerting much effort in identifying strategies to manage demanding business environments, employees have become more driven by their own career needs than by organizational goals. Therefore, it is not surprising that scholars have become increasingly interested in examining how organizational goals can be accomplished through employees’ career attitudes and behaviors (e.g., Fleisher et al., 2014; Dobrev and Merluzzi, 2018).

Recognizing the necessity of this new research approach reveals an important gap in the contemporary career literature that must be further explored and addressed. As part of the contemporary discourse about careers, we know, for example, that organizations can benefit from employees’ career mobility, which brings novel social and human capital (Dobrev and Merluzzi, 2018). We also know that employees’ career competencies, including their career motivation, knowledge, and network, can benefit organizations by strengthening the organizational core competencies of *culture, capabilities*, and *connections* (Fleisher et al., 2014). However, little is known about whether and how employees’ ambitions to advance in their careers benefit organizations. This may be partly owing to the negative connotations of ambition, which has been largely associated with individualistic cravings for power and self-interested behavior (Larimer et al., 2007; Pettigrove, 2007). In the present study, we refer to career-related ambitions as *career aspirations* and acknowledge that such aspirations indeed trigger self-interested behavior. Employees with career aspirations in particular proactively engage in skill-development behaviors and networking behaviors and proactively seek career consultation solely to plan for their careers (Strauss et al., 2012). Despite the self-interested career focus, some of these behaviors (i.e., skill-development and networking behaviors) could potentially positively influence important organization-level outcomes (e.g., Li et al., 2017; Shin and Konrad, 2017); hence, we believe that employees with career aspirations will benefit their employing organizations through their self-interested career behaviors. As this is the first study to focus on understanding the benefits that career aspirations might offer to organizations, we make an important contribution to the contemporary career literature. Next, we aim to highlight the important role that managers play in enhancing the positive effects of employees’ career aspirations on organizations. In line with a recent argument, we believe that although employees are capable of exercising control over their careers, organizations must still support them in achieving their career goals by providing them with human resource (HR) practices or organizational support programs (Jung and Takeuchi, 2018). More specifically, we argue that an empowering work design acts as a high-performance practice (Van De Voorde and Beijer, 2015), providing employees with the opportunities to fulfill their career aspirations, which in turn increases employees’ motivation to address organizational needs. In our understanding of such a work design, we refer to Grant and Parker (2009) work design perspective, which posits that *proactive* and *relational* aspects of work are the key characteristics of contemporary jobs. Specifically, the authors argue that due to increases in uncertainty and dynamism in work environments, it has become increasingly important for employees to be proactive and to take charge in anticipating future work needs and addressing them. Employees must also be more active in developing work relationships since interdependence and interactions with coworkers, customers and other stakeholders have increased (Grant and Parker, 2009). As relationship building and taking charge behaviors are both considered to also be proactive career behaviors (e.g., Fuller and Marler, 2009; Tschopp et al., 2016), we are the first to contribute to Grant and Parker (2009) work design perspective and to the HRM literature by demonstrating that contemporary work demands create opportunities for employees to address personal career and organizational needs. To explain our argument from a theoretical perspective, we follow recent work (e.g., Su et al., 2018) employing the Ability, Motivation and Opportunity (AMO) model and social exchange theory to explain why empowering HR practices could trigger employees to reciprocate by contributing to organizational performance. First, the AMO model states that high-performance work practices such as *work designs* are aimed at empowering employees to perform by enhancing their job-related abilities (A), by increasing their motivation levels (M) and by creating opportunities for them to positively contribute to the organization (O) (Appelbaum et al., 2000). We argue that Grant and Parker (2009) contemporary work design perspective not only enhances performance but also assists employees in fulfilling their career aspirations. For example, as we argue, the *proactive aspect* of work requires employees to be proactive by engaging in taking charge behaviors (Morrison and Phelps, 1999), which creates opportunities for them to enhance their job-related abilities and to develop career self-management skills. Then, the *relational aspect* of work provides employees with opportunities to develop *instrumental* and *developmental* relationships, affording them access to important resources that are needed to perform effectively and to progress in their careers (Higgins and Thomas, 2001). Second, consistent with the argument of social exchange theory and recent work (e.g., Sung et al., 2017; Su et al., 2018), we argue that employees will feel the need to reciprocate favorable organizational treatment with positive behaviors to benefit their organization (Whitener, 2001). Specifically, we argue that they will be motivated to utilize their job-related abilities and will engage in taking charge behavior to contribute to their organizations’ *capabilities*. We also argue that they will be motivated to strengthen their organization’s *connections* by utilizing their instrumental and psychosocial relationships to obtain important resources for the good of their organization. Simply stated, we thus propose that the proactive and relational aspects of work are the mechanisms through which employees with career aspirations contribute to organizations.

## Theory and Hypotheses

### Fulfilling Employees’ Career Aspirations: Opportunities Provided by Contemporary Work Design

There is a growing number of papers noting that the design of contemporary work is increasingly changing (Wegman et al., 2018). While aspects of work such as autonomy are still very relevant, more papers are suggesting that other aspects of work, such as proactivity, are becoming increasingly relevant in the contemporary workplace (Grant and Parker, 2009). This *proactive* aspect of work requires that employees adapt to dynamic jobs and contribute to shaping these jobs by introducing changes in the way that work is conducted (Morrison and Phelps, 1999; Frese and Fay, 2001).

To comply with this proactive nature of work, we argue that employees will be motivated to take charge at work, which is a form of proactive behavior that focuses on individual contributions to positive change with respect to the execution of work in organizations (Morrison and Phelps, 1999). By doing so, individuals need to actively seek out new information, identify opportunities for improvements and take action to improve work (Fuller et al., 2006). Therefore, engaging in taking charge behavior offers employees opportunities to gain new knowledge, to develop skills, and also to enhance their abilities, which are needed to perform successfully in higher positions in the future (Metz, 2004). Moreover, as other scholars have argued, proactive individuals who take charge at work will most likely also be better able to take charge of their careers (Fuller and Marler, 2009) and thus better achieve their career goals. Engaging in taking charge behavior could also directly open doors to better career opportunities, as the behavior signals strong leadership potential (Fuller et al., 2007). Considering these points together, we argue that employees with career aspirations will be motivated to take charge at work since doing so supports them in achieving their work and career goals. Therefore, we propose the following:

H1: Employees’ career aspirations will be positively associated with their taking charge behaviors.

Second, according to Grant and Parker (2009), we have also experienced changes in the social context at work, because teams are being used to complete work more frequently than in the past (Osterman, 2000). With this trend, employees are required to coordinate their work within their teams, as well as beyond their teams’ boundaries, with individuals and teams from different departments and organizations (Howard, 1995; Mohrman et al., 1995; Griffin et al., 2007). With the growing service sector, employees have also become responsible for addressing the needs of more demanding stakeholders (e.g., Schneider and Bowen, 1995; Batt, 2002; Grant and Parker, 2009), which requires that employees also proactively develop relationships outside their organizations. In other words, internal and external relationships have become more widespread and important in contemporary jobs.

This *relational* aspect of work offers opportunities for employees with career aspirations to develop relationships with influential individuals from inside and outside their employing organizations who can help them to progress and advance in their careers. Such relationships are called *instrumental relationships* and are especially beneficial for careers because they provide employees with important job-related information and resources (Higgins and Thomas, 2001). Indeed, research has demonstrated that being connected to influential individuals offers many career-related benefits, such as salary growth (Wolff and Moser, 2009), higher supervisor-rated promotion evaluations (Lin and Huang, 2005; Huang, 2016), more job-related knowledge (Forret and Dougherty, 2004), and more external job offers (Wolff and Moser, 2010). Therefore, we propose that employees with career aspirations will develop and use their instrumental relationships because such relationships can assist them in achieving their work and career goals.

H2: Employees’ career aspirations will be positively associated with their instrumental relationships.

Additionally, we also propose that employees with career aspirations will be motivated to develop *psychosocial* relationships at work. Such relationships are developed to gain emotional and appraisal support during one’s career and include role modeling, counseling and friendship (Higgins and Thomas, 2001). When employees set high career goals, they can have an unrealistic approach to career expectations, which could result in work-related stress (Van Emmerik et al., 2005), especially if they are not able to fulfill their personal expectations. Therefore, such employees are likely to be in need of role models who can offer them psychosocial support and help them reduce stress and other negative work-related outcomes such as exhaustion or burn-out. For instance, through psychosocial relationships, mentors can enhance employees’ sense of competence and self-esteem (Seibert, 1999; Ragins and Kram, 2007). They can also enhance employee resilience, an ability that is considered to be effective in maintaining employee well-being during high-pressure work situations (Ferris et al., 2005; Kao et al., 2014). In difficult times, such positive outcomes can motivate employees to pursue their career ambitions. Therefore, we formulate the following:

H3: Employees’ career aspirations will be positively associated with their psychosocial relationships.

### Employees’ Contributions to Organizations: The Roles of Proactive and Relational Aspects of Contemporary Work

Based on the AMO model, we employed Grant and Parker’s (2009) work design perspective to argue that it supports employees in fulfilling their career aspirations by developing their abilities, enhancing their motivation to pursue their career ambitions and by offering more and better career opportunities. Following social exchange theory, we now argue that employees with career aspirations will feel a sense of obligation to reciprocate by contributing positively to organizational *capabilities* and *connections*. As previously explained, we argue that the *proactive* and *relational* nature of work will provide employees with the opportunities to do so. Organizational capabilities refer to the knowledge and skills that are embodied in organizational activities (Quinn, 1992) and are strengthened when employees accumulate new skills and capabilities and utilize them for the good of the organization (Teece et al., 1997; Khapova et al., 2009; Wood et al., 2012). As previously argued, when employees take charge at work, they will most likely gain new knowledge and develop better skills; therefore, these employees will be better able to develop strategies to render work processes more effective and efficient (Wright et al., 1994) and to enhance productivity (Shin and Konrad, 2017). In line with this argument, Wu et al. (2014) found that new ideas and approaches facilitate product development and better ways of doing things in the workplace. Similarly, Yang et al. (2016) provide relevant evidence by showing that, when frontline bank employees creatively apply their knowledge to service procedures, they can help achieve innovation and enhance overall service performance. Therefore, we propose the following:

H4: Employees’ taking charge behaviors will be positively associated with their contribution to organizational capabilities.

Organizational connections, then, refer to the external contacts of organizations, such as suppliers, customers, alliances, and other stakeholders (Quinn, 1992). Organizational connections are likely to be strengthened when employees utilize their personal contacts to benefit their employing organizations (Khapova et al., 2009). Su et al. (2009) demonstrate, for example, that employees can acquire information from their personal relationships with customers and utilize it to contribute to positive organizational outcomes, such as product innovation. In a similar vein, Heirati et al. (2013) provide evidence that overall firm performance can be enhanced when employees utilize important market-related knowledge obtained from their personal ties with other firms, governmental officials and academic institutions. Consistent with the latter point, a recent study of Chinese firms in the manufacturing sector indicates that political and innovative economic stakeholders positively influence a firm’s innovative performance (Li et al., 2017), likely because employees can obtain information about important government regulations and innovation policies from their personal ties with political stakeholders and utilize this information to enhance their organizations’ innovative performance. Similarly, employees can obtain information from their relationships with competitors, suppliers, and buyers and utilize this information to develop better products and services for their employing organizations (Li et al., 2017). Therefore, the following two hypotheses are set:

H5: Employees’ instrumental relationships will be positively associated with their contribution to organizational connections.
H6: Employees’ psychosocial relationships will be positively associated with their contribution to organizational connections.

### Mediating Effects

We have argued that employees’ career aspirations are positively associated with their taking charge behaviors and instrumental and psychosocial relationships and that the latter strengthen organizational capabilities and connections. Building on these points, we also propose that the proactive and relational aspects of work are mechanisms through which employees’ career aspirations add value to organizations. This proposal is consistent with recent findings demonstrating that antecedents of firm performance are indirectly associated with actual firm performance through employees’ capabilities and behaviors (e.g., Shanker et al., 2017; Ashford et al., 2018). It is also in line with recent studies demonstrating that employees positively contribute to organizations through the resources that they obtain from their networks (e.g., Liu et al., 2017; Jiang et al., 2018). Therefore, the following three hypotheses are set:

H7: Taking charge behavior mediates the relationship between employees’ career aspirations and their individual contribution to organizational capabilities.
H8: Instrumental relationships mediate the relationship between employees’ career aspirations and their individual contribution to organizational connections.
H9: Psychosocial relationships mediate the relationship between employees’ career aspirations and their individual contribution to organizational connections.

**FIGURE 1:**
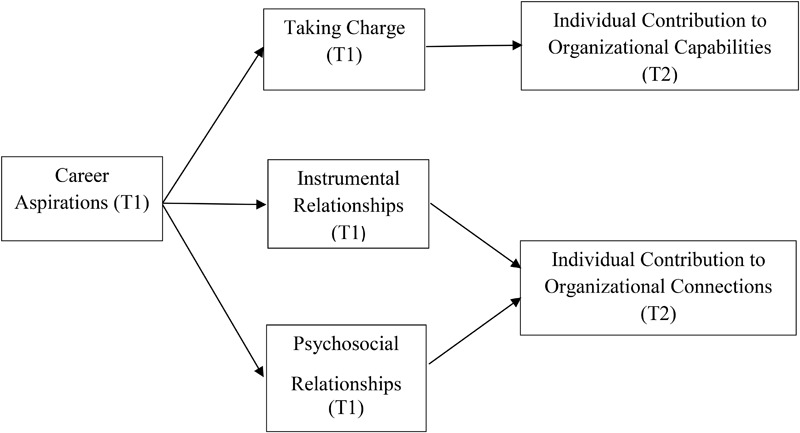
Research model.

Figure 1 provides an overview of all hypotheses.

## Methods

### Participants and Procedures

This study was initiated at a public university in the Netherlands. A web-based survey covering concepts such as work attitudes, career-related behaviors, and background factors was sent to a total of 4,880 business and economics graduates. The survey was sent at two time points with a 1-year time lag, in line with the suggestions of other scholars who argue that employee behaviors in work contexts should be examined with a 1-year time lag (Koys, 2001). Specifically, at time 1, the university was provided with a preliminary Microsoft Excel spreadsheet consisting of contact information from 2,000 graduates. At time 2, the contact information of the alumni group was completed; hence, the survey could be sent to an additional 2,880 potential candidates. At time 1, 558 graduates returned completed surveys, resulting in a response rate of 28%. At time 2, the response rate was 11%, since only 555 graduates returned completed surveys. The final sample (*N* = 181) consists only of participants who had completed the survey at times 1 and 2, who had graduated at least 1 year before the first survey was sent and who were employed at times 1 and 2. Thus, aside from normal attrition, the drop-outs were retirees, unemployed graduates and those not interested in participating in the project. The final sample (*N* = 181) consists of graduates holding job positions in different professional fields and industries such as management consultancy, HR management, accounting, and finance. Seventy-seven percent of the total sample was male, and the respondents were predominantly of Dutch nationality, with an average age of 37.7 years old (at time 1).

### Measures

Unless stated otherwise, abbreviated versions of the original scales were used, consisting solely of items fitting conceptually in the definitions of our study variables. In line with prior research using abbreviated versions of original scales (e.g., Leijten et al., 2015), we determined whether exploratory factor analysis revealed sufficient factor loadings for the selected items. The factor loadings ranged from 0.50 to 0.75 and could be considered sufficient for our sample size (*N* = 181) (Hair et al., 2010; Leijten et al., 2015). Additionally, the Cronbach’s alphas demonstrated that all of the scales were reliable (α > 0.77).

#### Career Aspirations

Five items from a scale developed by Gray and O’Brien (2007) were used to measure career aspirations. Example items include “I hope to become a leader in my career field,” “When I am established in my career, I would like to manage other employees,” and “I hope to move up through any organization or business in which I work” (1 = “strongly disagree” and 5 = “strongly agree”) (Baroudi, 2016, p. 88). The item scores were averaged to form total scores for career aspirations (T1 α = 0.77; T2 α = 0.78).

#### Taking Charge

Participants responded to Morrison and Phelps (1999) 10-item scale measuring taking charge. The items prefaced with “In my job, I often…” read as follows: “…try to adopt improved procedures for performing my job”; “…try to correct a faulty procedure or practice”; and “…try to implement solutions to pressing organizational problems.” A five-point scale was used, ranging from 1, “I strongly disagree” to 5 “I strongly agree,” with an option of “non-applicable” (Baroudi, 2016, p. 88). The item scores were averaged to form total scores for taking charge (T1 α = 0.91; T2 α = 0.91).

#### Instrumental Relationships

To measure instrumental relationships, seven items were used from a scale developed by Higgins (2001). The items prefaced with “In my network of relationships, I have people who…” read as follows: “…provide useful experiences for me professionally”; “…provide me with opportunities that benefit me professionally”; and “…open doors for me professionally” (1 = “not at all” and 5 = “to a great extent”) (Baroudi, 2016, p. 88). The item scores were averaged to form total scores for instrumental relationships (T1 α = 0.92; T2 α = 0.92).

#### Psychosocial Relationships

To measure psychosocial relationships, six items were used from Higgins and Thomas (2001) scale. The items prefaced with “In my network of relationships, I have people who…” read as follows: “…coach me on difficult work-related issues”; “…counsel me on work- and non-work-related issues”; and “…frequently act as supporters” (1 = “not at all” and 5 = “to a great extent”) (Baroudi, 2016, p. 88). Again, the item scores were averaged to form total scores for psychosocial relationships (T1 α = 0.90; T2 α = 0.89).

#### Individual Contribution to Organizational Capabilities

The participants also completed Khapova et al. (2009) (in Fleisher et al., 2014), eight-item scale measuring individuals’ contributions to organizational capabilities. Example items include “I try to use my external experiences to improve existing ways of doing things in my organization” and “I try to use what I learn outside my organization in my work” (Baroudi, 2016, p. 89). The respondents used a five-point Likert scale ranging from 1 “I strongly disagree” to 5 “I strongly agree.” The item scores were averaged to form total scores for individuals’ contributions to organizational capabilities (T1 α = 0.80; T2 α = 0.81).

#### Individual Contribution to Organizational Connections

The participants completed five items from Khapova et al. (2009) scale (in Fleisher et al., 2014). The items prefaced with “In my work…” read as follows: “…my external contacts assist me in work-related problem solving” and “I utilize knowledge generated from my external professional activities” (1 = “strongly disagree” and 5 = “strongly agree”). Item scores were averaged to form total scores for individual contributions to organizational connections (T1 α = 0.84; T2 α = 0.83).

#### Control Variables

Following the recommendations of Carlson and Wu (2012), we looked for demographic variables that have significant correlations with our study variables to include them as control variables. Only gender and age were found to meet the requirement; therefore, other demographic variables were omitted from our statistical analyses. Indeed, in prior studies, gender was found to have associations with career aspirations such that fathers were more likely to consider promotion to be the most important career aspiration, compared to mothers (van der Horst et al., 2014). Age was also found to influence career advancement motives in another study such that motives were found to decrease when employees’ age increased (Kooij et al., 2011). Older employees were also found to build work relationships to help others, rather than utilize the relationships to fulfill career needs (Kooij et al., 2011). Age was also found to positively influence the quality of organizational performance in prior work (Backes-Gellner et al., 2011). Finally, including gender and age as control variables is also consistent with prior research examining the effects of antecedents on taking charge behaviors (Morrison and Phelps, 1999; Burnett et al., 2015). Gender was recorded as a dummy variable (i.e., male = 1 and female = 2) in our statistical analyses.

### Statistical Analysis

To examine the validity of the measurement model, we ran a confirmatory factor analysis in which we included all of our main variables. The results demonstrated a good model fit (χ^2^ = 1166.08, *df* = 764, RMSEA = 0.05, NFI, CFI = 0.96) (e.g., Hu and Bentler, 1999; Hooper et al., 2008), indicating that each measure had a good level of convergent and discriminant factorial validity. Given that all of the measures in the model were self-reported from a single survey, we also tested for common method variance. Following the recommendation of Podsakoff et al. (2012), the procedure involved comparing alternative models as well as adding a latent common method variable. In this case, the baseline proposed model also obtained a better fit, suggesting that common method variance did not inordinately influence our results. To test the direct and mediated effects formulated in our hypotheses, we used Process (version 2.15) in IBM SPSS Statistics software, version 24. Process assesses mediation effects based on bias-corrected confidence intervals obtained from 5,000 bootstrapped estimates of each required path (Hayes, 2009). To investigate the effects of the mediators (i.e., taking charge, instrumental, and psychosocial relationships) on the relationship between career aspirations and individuals’ contribution to organizational capabilities and connections, we included the mediators of T1 in our analyses. This approach is in line with prior work investigating mediation effects with two waves (e.g., Solove et al., 2015). Finally, in line with prior longitudinal research, we included the control variables of T1 in all of our analyses (i.e., gender and age) (Tims et al., 2015).

## Results

Table 1 presents the descriptive statistics and correlations for all of the study variables. The study variables were all positively correlated, except for taking charge (T1 + T2), which was not related to instrumental and psychosocial relationships (T1). The control variable age was negatively related to gender and instrumental and psychosocial relationships (T1) but positively related to taking charge (T2), individual contribution to organizational capabilities (T1 + T2) and individual contribution to organizational connections (T1 + T2). Gender was negatively related to individual contribution to organizational capabilities (T1) and individual contribution to organizational connections (T2).

**Table 1:** Descriptive statistics and correlations among the study variables.

Variable	Mean	*SD*	1	2	3	4	5	6	7	8	9	10	11	12	13	14
(1) Age	37.67	9.51														
(2) Gender	1.22	0.42	-0.28^∗∗^													
(3) Career aspirations T1	3.72	0.66	-0.13	-0.07	(0.77)											
(4) Career aspirations T2	3.65	0.72	-0.10	-0.05	0.66^∗∗^	(0.78)										
(5) Taking charge T1	3.94	0.56	0.13	-0.13	0.44^∗∗^	0.34^∗∗^	(0.91)									
(6) Taking charge T2	4.01	0.55	0.23^∗∗^	-0.07	0.30^∗∗^	0.28^∗∗^	0.63^∗∗^	(0.91)								
(7) Instrumental relationships T1	2.96	0.83	-0.20^∗∗^	-0.03	0.23^∗∗^	0.26^∗∗^	0.10	0.12	(0.92)							
(8) Instrumental relationships T2	2.98	0.85	-0.14	-0.03	0.36^∗∗^	0.39^∗∗^	0.29^∗∗^	0.30^∗∗^	0.59^∗∗^	(0.92)						
(9) Psychosocial relationships T1	3.14	0.80	-0.27^∗∗^	0.03	0.27^∗∗^	0.28^∗∗^	0.11	0.12	0.82^∗∗^	0.50^∗∗^	(0.90)					
(10) Psychosocial relationships T2	3.22	0.76	-0.14	0.06	0.26^∗∗^	0.36^∗∗^	0.28^∗∗^	0.30^∗∗^	0.49^∗∗^	0.75^∗∗^	0.53^∗∗^	(0.88)				
(11) Individual contribution to organizational capabilities T1	3.86	0.45	0.26^∗∗^	-0.18^∗^	0.38^∗∗^	0.32^∗∗^	0.39^∗∗^	0.43^∗∗^	0.33^∗∗^	0.42^∗∗^	0.25^∗∗^	0.26^∗∗^	(0.80)			
(12) Individual contribution to organizational capabilities T2	3.87	0.50	0.28^∗∗^	-0.14	0.25^∗∗^	0.28^∗∗^	0.25^∗∗^	0.42^∗∗^	0.24^∗∗^	0.43^∗∗^	0.18^∗^	0.30^∗∗^	0.62^∗∗^	(0.86)		
(13) Individual contribution to organizational connections T1	3.41	0.73	0.29^∗∗^	-0.11	0.35^∗∗^	0.28^∗∗^	0.20^∗∗^	0.23^∗∗^	0.38^∗∗^	0.44^∗∗^	0.28^∗∗^	0.30^∗∗^	0.68^∗∗^	0.50^∗∗^	(0.84)	
(14) Individual contribution to organizational connections T2	3.63	0.65	0.30^∗∗^	-0.18^∗^	0.27^∗∗^	0.25^∗∗^	0.20^∗∗^	0.36^∗∗^	0.26^∗∗^	0.48^∗∗^	0.20^∗∗^	0.33^∗∗^	0.57^∗∗^	0.74^∗∗^	0.66^∗∗^	(0.83)

*Sample sizes for the correlations range from 174 to 181. The control variables age and gender are from time 1. Alpha internal consistency reliability coefficients appear on the main diagonal. ^∗^*p* < 0.05, ^∗∗^*p* < 0.01.*

Results of the mediation analyses are reported in Tables 2, 3. Support was found for Hypothesis 1, which stated that employees’ career aspirations are positively related to employees’ taking charge behavior (*b* = 0.42, *p* < 0.01). Hypothesis 2, which stated that employees’ career aspirations are positively related to instrumental relationships, was also supported (*b* = 0.25, *p* < 0.01). The results also supported Hypothesis 3, which stated that employees’ career aspirations are positively related to psychosocial relationships (*b* = 0.28, *p* < 0.01). Hypothesis 4, which stated that employees’ taking charge behavior is positively related to employees’ contribution to organizational capabilities was not supported (*b* = 0.09, *p* > 0.05). Hypotheses 5 and 6, which stated that employees’ instrumental and psychosocial relationships are positively related to employees’ contributions to organizational connections, were supported (*b* = 0.22, *p* < 0.01) and (*b* = 0.19, *p* < 0.01). Moreover, although not formulated as hypotheses, Table 2 shows that employees’ career aspirations are positively related to the dependent variables of individual contribution to organizational capabilities (*b* = 0.18, *p* < 0.01) and connections (*b* = 0.25, *p* < 0.01). Table 3 shows the results of our mediation hypotheses 7–9. Hypothesis 7, which stated that employees’ taking charge behavior mediates the relationship between employees’ career aspirations and their individual contribution to organizational capabilities, was not supported. The indirect effect of taking charge behavior (0.04) is not significant within a 90% confidence interval of -0.0390 to 0.1152. Support was found for Hypothesis 8. The indirect effect of employees’ instrumental relationships (0.05) on the relationship between employees’ career aspirations and their individual contribution to organizational connections is significant within a 90% confidence interval of 0.0199 to 0.1094. Finally, the indirect effect of employees’ psychosocial relationships (0.05) on the relationship between employees’ career aspirations and their individual contribution to organizational connections (i.e., Hypothesis 9) was also significant within a 90% confidence interval of 0.0209 to 0.1100.

**Table 2:** Results for direct relationships.

	Taking charge	Instrumental relationships	Psychosocial relationships	Individual contribution to organizational capabilities	Individual contribution to organizational connections
Age	0.01^∗∗^	-0.02^∗∗^	-0.02^∗∗^	0.02^∗∗^	0.03^∗∗^
Gender	-0.07	-0.14	-0.05	-0.03	-0.08
Career aspirations	0.42^∗∗^	0.25^∗∗^	0.28^∗∗^	0.18^∗∗^	0.25^∗∗^
Taking charge				0.09	
Instrumental relationships					0.22^∗∗^
Psychosocial relationships					0.19^∗∗^
Total *R*^2^	0.24^∗∗^	0.09^∗∗^	0.13^∗∗^	0.17^∗∗^	0.26^∗∗^

*Values are based on the entry of all of the variables listed at each step. We controlled for the variables of age and gender of T1 in all of our analyses. The independent variable career aspirations, the mediators taking charge and instrumental and psychosocial relationships are of T1. The dependent variables of individual contribution to organizational capabilities and connections are of T2. Regression coefficients are unstandardized values. ^∗^*p* < 0.05, ^∗∗^*p* < 0.01.*

**Table 3:** Mediation results with bootstrapping.

Mediation effect	Point estimate	Lower confidence interval	Upper confidence interval
Taking charge	0.04	-0.0390	0.1152
Instrumental relationships	0.05	0.0199	0.1094
Psychosocial relationships	0.05	0.0209	0.1100

*All of the values were tested for significance using 90% bias-corrected confidence intervals from 5,000 bootstrapped intervals. We controlled for the variables age and gender of T1 in all our mediation analyses. The independent variable of career aspirations and the mediators of taking charge and instrumental and psychosocial relationships are of T1. The dependent variables of individual contribution to organizational capabilities and connections are of T2.*

## Discussion

Our study aimed to develop and examine a theoretical model (Figure 1) of (1) the link between employees’ career aspirations and their contributions to organizations; and (2) the mechanisms (i.e., proactive and relational aspects of contemporary work) underlying this link. The study results confirm that employees with career aspirations contribute to strengthening: (a) organizational capabilities; and (b) organizational connections through their instrumental and psychosocial relationships. Interestingly, although career aspirations were positively associated with taking charge, we did not find that taking charge predicts employees’ individual contributions to organizational capabilities. Hence, a mediating effect of taking charge on the direct relationship between employees’ career aspirations and their individual contributions to organizational capabilities was also not found. Based on these results, we make a number of significant contributions.

The first contribution concerns the link between employees’ career aspirations and their contribution to organizations. While recent studies have demonstrated how employees’ career mobility benefits organizations (Dobrev and Merluzzi, 2018) and how organizational career management, as well as individual career investments, are beneficial for firm performance (Crook et al., 2011; De Vos and Cambré, 2017), we extend support for this reasoning by demonstrating that employees’ career aspirations also strengthen organizational capabilities and connections. To date, research has examined career aspirations of high school and university students to understand and influence their future career paths (e.g., Schoon and Polek, 2011; Kitchen et al., 2017). For instance, more recently, insufficient student interest in STEM careers has prompted scholars to examine students’ career aspirations and to identify effective strategies to inspire more students to pursue STEM careers (e.g., Kitchen et al., 2017; Holmes et al., 2018). Contributing to this recent work, our results suggest that career aspirations are not only useful for influencing students’ future careers but are also an important force for achieving positive organizational outcomes.

Our findings also contribute to the understanding of the proactive and relational aspects of contemporary work according to Grant and Parker’s (2009) work design perspective and to the HRM literature focusing on demonstrating the effects that high-performance practices have on employees’ willingness to contribute to organizations (e.g., Jeong and Shin, 2017; Shin and Konrad, 2017). We demonstrate that the relational aspect is an important mechanism explaining the positive relationships between employees’ career aspirations and their contributions to organizational connections. Interestingly, although findings show that employees with career aspirations engage in more taking charge behaviors, this fact does not subsequently lead to a contribution to organizational capabilities. We assume that this finding can be partly ascribed to the behavior itself, as taking charge is often considered to be a risky behavior and, hence, requires careful nurturing (Burnett et al., 2015). Employees could engage in taking charge behavior to fulfill their career aspirations, but when they decide to utilize their taking charge experience to contribute to their employing organization, they could fear that their manager and even the top management may perceive their effort as threatening rather than positive. Therefore, we recommend future research to examine the influence of organizational support on the mediated relationship among employees’ career aspirations, their taking charge behaviors and their individual contribution to organizational capabilities. By doing so, special attention should be paid to employee perceptions of fairness regarding the distribution of other HR practices (next to work design) since doing so might also influence employees’ willingness to contribute to organizational performance (Vanhala and Ritala, 2016).

Finally, returning to our findings regarding the relational aspect, we find this aspect to be an important mechanism explaining the positive relationship between employees’ career aspirations and their contributions to organizational connections. Specifically, we find that employees’ career aspirations are positively associated with their instrumental and psychosocial relationships, which are in turn both beneficial to organizations. These findings are consistent with prior work demonstrating that employees can obtain important resources from their relationships and utilize them for the good of their company (e.g., Su et al., 2009; Heirati et al., 2013; Li et al., 2017). However, we extend this work by specifying the type of employees that are likely to do so (i.e., career ambitious employees). Moreover, we also demonstrate that, although employees develop different types of relationships to achieve different career goals, such relationships appear to involve a more unified role for achieving positive organizational outcomes. Consistent with recent work on career relationships (Van Vianen et al., 2018), we thus show that it is worthwhile to focus on examining the effects of career relationships at distinguished organizational levels (i.e., individual and organization levels).

### Study Limitations and Future Research Directions

Despite the significant contributions, we acknowledge that this study has some limitations that must be addressed in future research. For instance, we used two waves of data to examine mediation effects in relationships in which the independent variable and mediators are from time 1, and the dependent variables are from time 2. According to other scholars, this process means that the results cannot be interpreted causally and that the real direction of effects is possibly counter to the hypothesized effects (Holman et al., 2012). A natural follow-up to this study would be to examine similar mediation predictions using a design in which the independent variable, mediators and dependent variables are from different time points. Three waves of data would allow for time 1 data to be used for the predictor (X), time 2 data to be used for the mediator (M), and time 3 data to be used for the outcome variable (Y) (Rose et al., 2004), thus making it possible to better distinguish between causes and effects (Cole and Maxwell, 2003; Baroudi et al., 2017). Another limitation concerns the reliance on self-reported data in our measurements. Measuring relationships between variables with self-reported surveys only could result in inflation due to mono-method bias or common method variance (Podsakoff et al., 2012; Baroudi, 2016). We minimized concerns about inflated relationships in our study because we tested for common method variance following the recommendation of Podsakoff et al. (2012). The results suggested that common method variance does not inordinately affect the study findings. Nevertheless, more objective measures could be used in future studies examining similar predictions to better ensure that the results are unbiased (Holman et al., 2012). A third limitation of our study concerns our statistical analytical approach. Researchers have suggested that techniques such as structural equation modeling (Jöreskog and Sörbom, 1993) might be more appropriate for testing mediation models with longitudinal data. However, as a limitation, such techniques require larger samples to observe significant changes in effects (e.g., Finch et al., 1997; Fan and Wang, 1998). To overcome weak mediation analyses and results (Dudley et al., 2004), we followed prior work and tested our mediation hypotheses using bias-corrected confidence intervals from 5,000 bootstrapped intervals (e.g., Baroudi et al., 2017). According to Williams and MacKinnon (2008) and Clifford et al. (2017), bootstrapping is a robust method for testing the effects of mediating variables. Nevertheless, we recommend that future researchers test our mediation predictions using structural equation modeling and a larger sample to augment our research results and conclusions.

### Practical Implications

A key practical implication of this study is that managers should utilize HRM practices effectively to motivate employees not only to follow their career aspirations but also to utilize their personal resources for the good of the organization. For instance, this study suggests that managers should aim to recruit career ambitious employees and retain them, as this group of employees contributes to making organizations strong by strengthening organizational capabilities and organizational connections. The work design of such employees also plays an important role because the relational aspect of work was found to benefit career ambitious employees and their employing organizations. However, attention should be devoted to the proactive aspect of work, as employees with career aspirations do engage in taking charge behavior but do not use this experience to contribute to their organization’s capabilities. Therefore, we recommend that managers encourage employees to orient their taking charge behaviors toward benefiting the organization (Griffin et al., 2007), by for example rewarding employees’ proactive efforts and behaviors.

## Ethics Statement

For this study, ethics approval was not required per the Vrije Universiteit’s guidelines and national regulations. Nevertheless, the research was conducted in line with the *Research Ethics Regulations* of the School of Business and Economics of the Vrije Universiteit Amsterdam. All of the participants were voluntarily involved in the study. Emails were sent to the participants, in which the purpose and procedures of the study were described and in which their approval was asked for participation in the study. The online survey included a consent form with all of the necessary information that participants had to read and approve prior to proceeding with the survey. Moreover, the participants were allowed to withdraw from the study at any time. The participants who decided to withdraw from the study were not included in the final sample, and their data were destroyed.

## Author Contributions

SB was involved in conception and design of the study, in the data collection and analysis, and in the manuscript writing. SK was involved in conception and design of the study, the data collection and manuscript writing and approved the submitted version. CF was involved in the data collection and analysis. PJ was involved in the data analysis and approved the submitted version.

## Conflict of Interest Statement

The authors declare that the research was conducted in the absence of any commercial or financial relationships that could be construed as a potential conflict of interest.
